# Novel Hot Vapor Filter Design for Biomass Pyrolysis

**DOI:** 10.1021/acs.energyfuels.2c04285

**Published:** 2023-02-23

**Authors:** Christian Lindfors, Matti Nieminen, Tamer Alhalabi, Elmeri Pienihäkkinen, Joona Lahtinen, Anja Oasmaa

**Affiliations:** VTT Technical Research Center of Finland Ltd., VTT, P.O. Box 1000, Espoo FI-02044, Finland

## Abstract

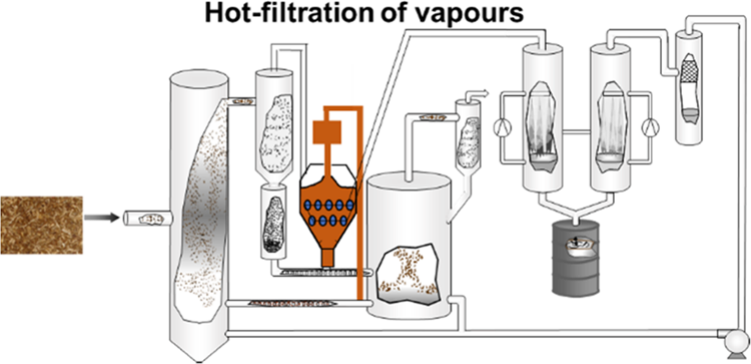

Fast pyrolysis is a mature technology for the conversion of solid biomass into a liquid intermediate, fast pyrolysis bio-oil (FPBO). FPBO has so far been used mainly as heating fuel, but the target is to use it for the production of sustainable fuels and chemicals in the future. In the pyrolysis process, inorganic materials (ash) from the biomass are mostly sequestered in char particles, which can be separated with cyclones. Small particles (<10 μm) escape the cyclones and condense with vapors. In bio-oils, these solid materials are unfavorable because they can cause erosion, corrosion, and blockages at injection nozzles in power generation systems and deactivate the catalyst in bio-oil upgrading. Hot vapor filtration using either moving bed or barrier filters has been tested on a small scale for the removal of fine particles from the pyrolysis vapors. The challenges with these filter types have been the increased pressure drop across the filter with time, inefficient solid removal, and loss in organics. A new hot vapor filter combining both a barrier filter and a moving bed filter was constructed and tested to overcome these problems. In this filter system, the filter elements are inserted in a vessel, where hot sand flows through to continuously remove the cake over filter candles. The filter was successfully tested with stem chips, contaminated wood, and forest residue for 6–8 h without any pressure increase. The organic liquid yield decreased in the best case only by 3 wt % using the lowest filtration temperature and shortest residence time. The oil properties were slightly affected by cracking of the sugar fraction, which decreased the oxygen content, microcarbon residue, and carbonyl content but increased the acidity. Only minor improvements in metal removal were seen due to the high detection limits of metal analysis.

## Introduction

Fast pyrolysis of clean wood is a mature technology with production facilities in Europe and Canada.^1^ Due to economic and environmental constraints, more sustainable and typically low-quality feedstocks should be used in the future. Also, more valuable products including fuels and chemicals should be produced to contribute to the net zero emission target by 2050.^2,3^ The use of more challenging feedstocks with a higher level of impurities together with the high-value end-user applications sets certain specifications for the quality of an intermediate, fast pyrolysis bio-oil (FPBO). Especially, inorganic impurities in the bio-oil are harmful because they settle at the bottom of the vessel in the form of sludge during bio-oil storage, cause erosion and corrosion, block injection nozzles in power generation systems, and form deposits on the surface of the catalyst, which changes its activity and shortens its lifetime, when FPBO is further upgraded.^4−7^ The most challenging components of ash are alkali and alkaline earth metals (AAEMs), especially K, which are catalytically active during pyrolysis.^8,9^

During biomass pyrolysis, most of the inorganics remain in the char, but smaller particles (<10 μm) escape the cyclones and condense with vapors. Some inorganic elements on char particles might further form bonds with organic volatiles, after which the composed molecule can be released in the vapor phase and end up in the bio-oil. This transfer of metals is however less common in pyrolysis and might only take place for few of the elements, especially Na and K.^10^ Methods to reduce the metal content in the bio-oil include washing of the feedstock before pyrolysis, filtration, or ion exchange of the FPBO and filtration of the hot vapors in the pyrolysis process. Alkali removal from the feedstock by washing with an acidic water mixture increases the organic liquid yield, but the lack of catalytically active elements in biomass might result in melting of especially the lignin onto heat carrier sand, causing bed agglomeration.^11,12^ An extra washing step for the feedstock before pyrolysis increases both the capital investment and operational cost of the process.^13^ Solid removal from the produced bio-oil by filtration is difficult because of the clogging of filters due to the sticky material in bio-oil. In addition to this, part of the metals from the solids may be leached to the bio-oil, and therefore, the filtration of the solids will not completely remove the metals.^14^ Metal ions in FPBO can be removed with an ion exchange process, but the adjustment of the kinematic viscosity of bio-oil to about 60 mm^2^/s or less is needed to make the removal at ambient temperature possible.^15^ Hot filtration of pyrolysis vapors has been reported to efficiently reduce inorganic material content in the bio-oil, but the main challenge with this technology has been how to maintain constant pressure in the system during operation.^16,17^

Hot filtration of pyrolysis vapors has been carried out using either barrier filters or moving bed filters. A barrier filter consists usually of a filter candle with a permeable wall and a closed and open end. During the filtration process, the raw gas flows through the wall of the filter and leaves from the open end of the candle. As the dust particles get separated, a dust cake builds up on the outer surface of the candle, which increases the pressure drop across the filter. When the pressure drop across the filter reaches a certain value, a reverse jet pulse of pressurized gas is injected in the opposite direction of the filtration flow to detach the filter cake. In the pyrolysis process, this filter cake is sticky and cannot therefore easily be removed from the surface of the filter by nitrogen pulsing.^16^ Controlled oxidation has also been tested for the removal of the char from the filter, but this procedure took up to 6–9 h to complete the regeneration. After controlled oxidation, residual ash remained on the filter cloth fibers, which may act as a catalyst and react with the pyrolysis vapors that pass through the filter to produce additional gases and char.^18^ A filter (wire mesh, pore size 5 μm) immersed in a fluidized bed reactor was tested at the University of Twente to overcome the operational problems related to the increase in pressure drop across the filter with time. Good process stability concerning temperature and pressure drop across the filter was achieved during a 2 h run. Sand and char were retained at the outside of the filter and the product yields were comparable to those carried out without the filter. The attrition of the filter element by the sand in a fluidized bed might be a challenge in a continuous commercial process.^19^

In a moving bed filter, particulates and pollutants are removed from the product vapors by passing the dirty gas through a bed of granulates. The granular flow is typically downward aided by gravity and controlled at the bottom by a rotating auger. In moving bed filters, the pressure build-up across the filter is not a problem. However, fine sand dust might be entrained with the gases, affecting the bio-oil solid content in a negative way.^20^

The bio-oil quality has been significantly improved after hot vapor filtration. Solid content below 0.01 wt % and alkali metal concentration below 10 ppm have been measured. Also, a significant increase in product quality due to lower viscosities and reduced aging of the filtered oil has been observed.^16−18^ Catalytic hydrotreatment experiments have also been carried out with a hot vapor filtered oil, and the results have been compared with unfiltered oil. The results show that even if the unfiltered oil was low in mineral content, there was a clear difference in deposit formation on the used catalyst between the filtered and unfiltered oil.^21^

The target of this project was to develop a new filter type to overcome the operational problems related to the increase in pressure drop across the filter and the decrease in oil yield. The new filter type was tested first with clean wood to optimize the process conditions and after that with contaminated wood and forest residue to evaluate the performance of metal removal in the filter.

## Experimental Section

### Feedstock Analyses

Stem chips, contaminated wood class B from Lassila and Tikanoja (L&T) in Finland, and forest residue were used as the feedstock for the pyrolysis experiments. Stem chips contained mainly pine and spruce and were used for the commissioning tests of the filter. Contaminated wood class B and forest residue were selected based on their availability and sustainability as potential feedstocks for the pyrolysis process in the future. Wood graded as B contains coated, lacquered, or otherwise chemically treated wood without halogenated organic compounds (for example PVC) and preservatives. Maximum 2 wt % mechanical contaminants such as cement or nails are allowed in class B wood and the annual averages of chlorine and heavy metal contents may not exceed the threshold values of virgin wood according to the solid biofuel standard (EN ISO 17225-1).^22^ Forest residue has a higher ash content compared to stem chips and contaminated wood and was therefore selected as the third feedstock to evaluate the metal removal in the filter.

Before the experiments, feedstocks were dried to a moisture content < 10 wt % and ground to a particle size of 0.5–1 mm. Proximate and ultimate analyses of the raw materials are presented in Table 1. Raw material analysis was done according to EN ISO methods.

**Table 1:** Proximate and Ultimate Analyses of Stem Chips, Contaminated Wood, and Forest Residue

origin	unit		stem chips	contaminated wood	forest residue
moisture	wt %	SFS-EN ISO 181343	7.5	8.0	6.8
volatiles	wt %, dry	SFS-EN ISO 18123	83.9	84.7	79.9
ash 550 °C	wt %, dry	SFS-EN ISO 18122	0.2	0.8	5.1
carbon	wt %, dry	SFS-EN ISO 16948	51.1	50.4	48.1
hydrogen	wt %, dry	SFS-EN ISO 16948	6.0	6.0	5.4
nitrogen	wt %, dry	SFS-EN ISO 16948	0.1	0.4	0.1
HHV	MJ/kg, dry	SFS-EN 18125	20.27	20.17	19.67
LHV	MJ/kg, dry	SFS-EN 18125	18.97	18.86	18.49
Cl	wt %, dry	SFS-EN ISO 16994	0.000	0.020	0.007
S	wt %, dry	SFS-EN ISO 16994	0.010	0.017	0.011

AAEM (alkali and alkali earth metal) contents in raw material alongside with other metals are presented in Table 2. Calcium is abundant in all feedstocks. Forest residue also contains a lot of silicon, sodium, potassium, and iron probably due to some soil mixed with the residues during harvesting. Analytical techniques such as inductively coupled plasma mass spectrometry (ICP-MS), inductively coupled plasma atomic emission spectrometry (ICP-AES), and oxygen bomb ion chromatography were used. Analytical techniques were conducted according to EN ISO standards (SFS-EN ISO 16967 (A), SFS-EN ISO 17294-2, SFS-EN ISO 11885, SFS-EN ISO 16994, SFS-EN 15408, and SFS-EN ISO 10304-1).

**Table 2:** Alkali and Alkaline Earth Metal Content in Stem Chips, Contaminated Wood, and Forest Residue Raw Material Analyzed on Dry Basis

	metals	unit	stem chips	contaminated wood	forest residue
AAEM	Na	mg/kg, dry	25	350	1900
	K	mg/kg, dry	450	500	3800
	Mg	mg/kg, dry	240	230	720
	Ca	mg/kg, dry	1000	1700	2500
other metals	Cr	mg/kg, dry	3	6	7
	Mn	mg/kg, dry	78	80	92
	Fe	mg/kg, dry	170	210	2300
	Cu	mg/kg, dry	33	6	4
	Zn	mg/kg, dry	41	58	29
	Si	mg/kg, dry	270	790	21 000
	Pb	mg/kg, dry	5	20	4
	P	mg/kg, dry	68	55	100

## Experimental Setup

**Figure 1:**
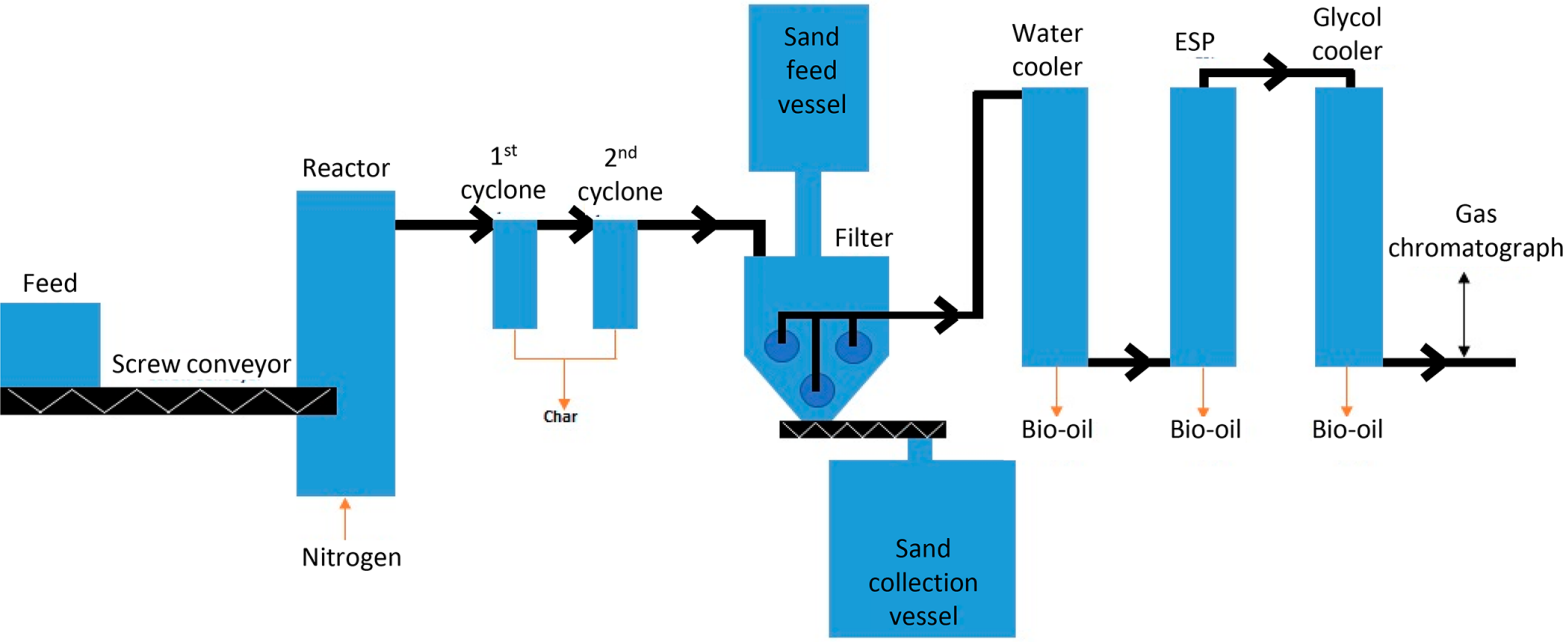
Schematic flow diagram of the bench-scale fast pyrolysis unit with the hot vapor filter.

Pyrolysis experiments were carried out using a bench-scale bubbling fluidized bed (BFB) unit (Figure 1).

The reactor diameter (*d*) is 52 mm, the height (*h*) is 570 mm, and heat for the pyrolysis is provided from outside with four different electrical heating elements, which enable temperature control through the whole reactor length. The reactor was operated under atmospheric pressure at 480–500 °C and with a gas phase residence time of 1 s. The temperature varied ± 5 °C during the experiments. The length of the experiment was 3–8 h. In the reactor, 300 g of white aluminum oxide (0.56–0.71 mm, ρ = 4000 kg/m^3^) was used as a bed material, which was fluidized by nitrogen. The char left after pyrolysis was separated from the gases with two cyclones.

**Figure 2:**
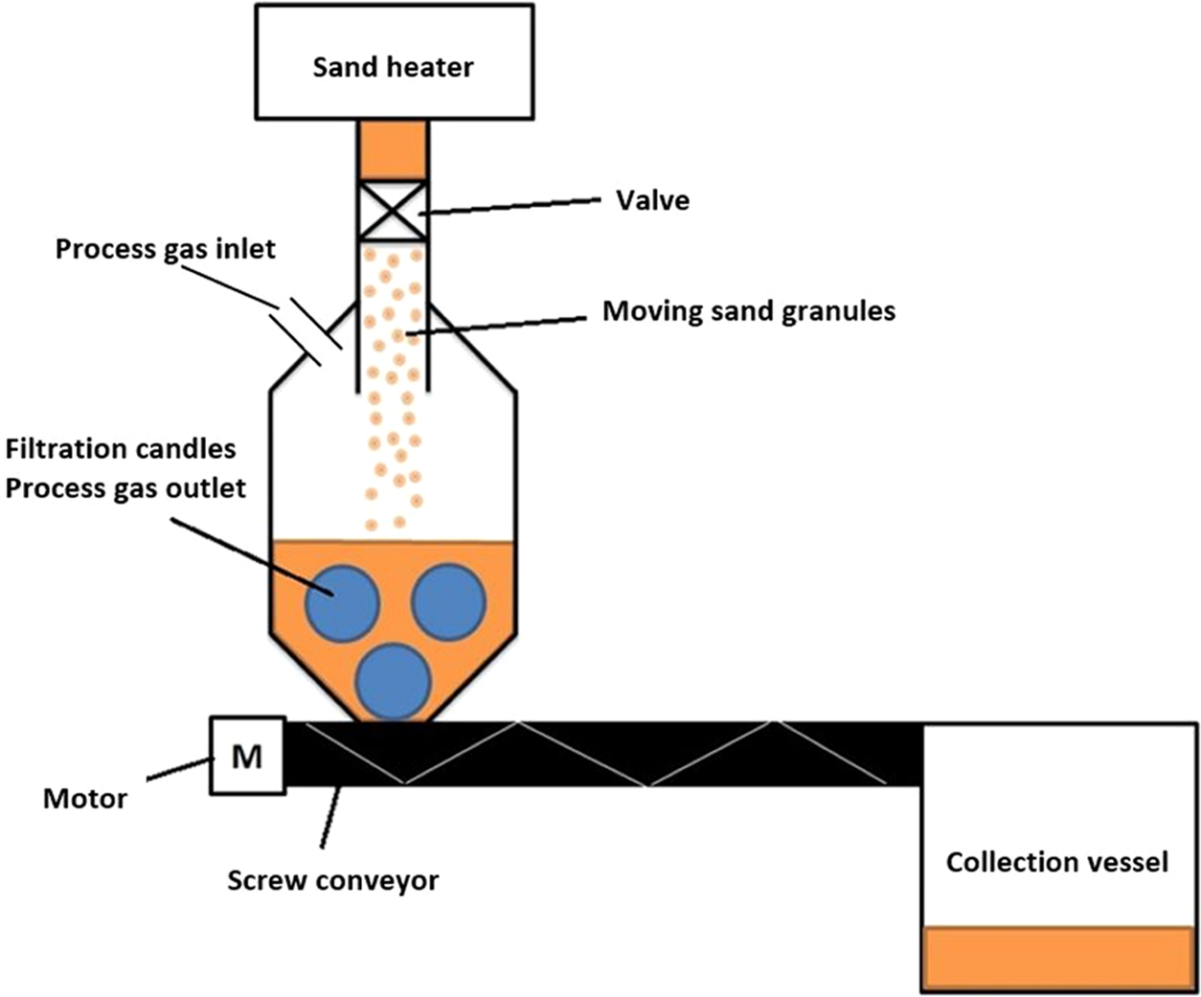
Hot vapor filter designed by VTT.^22^

After the cyclones, a hot gas filter was used for cleaning of the vapors before condensation. One experiment was carried out without the cyclones to investigate if the hot vapor filter could be located directly after the reactor. The operational principle of the new hot vapor filter developed at VTT is based on combining a barrier filter with a moving bed filter.^23^ Dia-Schumalith (10–20) ceramic filter candles Do/Di 60/40 mm, length 300 mm, and pore size 0.3 μm were ordered from Pall Corporation for the tests. The filter elements were inserted in a vessel, where hot quartz granules are flowing through, which assures continuous removal of cake over the filter candles and prevents the pressure increase across the filter (Figure 2). In the tests, sand particles of 0.2–1 mm were used. The open end of the filter element was affixed to a metallic tube sheet, which supported the element and served to separate the filtered gas from the unfiltered process stream and sand. Both the vessel and the connection tubes were trace-heated. The quartz sand granules were heated in a sand heater vessel to the desired filtration temperature before the experiment. The sand was drawn out of the filter vessel with a flow of 1.5 and 3 kg/h by a screw conveyor into a collection vessel. The design parameters selected for our filter at VTT were (1) maximum gas flow rate of 45 L/min NTP (normal temperature and pressure), (2) maximum filter temperature of 500 °C, and (3) face velocity of 1–4 cm/s. The maximum gas flow rate was calculated based on the amount of nitrogen used in the bench-scale unit for fluidization (38 L/min NTP) and the estimated amounts of produced gases and vapors from the feedstock (5 L/min NTP) using a feeding capacity of 0.8 kg/h. To achieve the desired face velocity of 1–4 cm/s, either 3, 2, or 1 filter candle was used in the tests. The length of the filter vessel was 30 cm, breadth 24 cm, and height 34 cm. The upper part of the vessel was constructed as a snapped cone with the sand tube going 5 cm into the vessel to form an empty space for the dirty gas above the sand. The filter candles were located 10 cm below the sand pipe to form a sand pile above the filters with a thickness of at least 3 cm. The volume of the filter vessel was 17.6 L and the amount of sand in the vessel was 11.8 L, using three filter candles.

**Figure 3:**
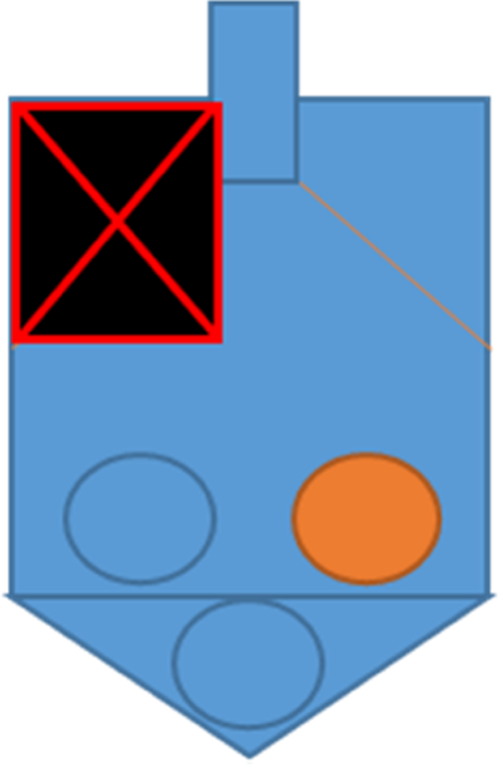
Modified filter with a metal plate inside the filter vessel.

Bio-oil yield in hot vapor filtration is dependent both on the filtration temperature and gas phase residence time. The highest bio-oil yield (10 wt % decrease in organics) reported by NREL has been obtained using the lowest filtration temperature, where condensation of the vapors on the filter candle does not occur, and with the shortest residence time.^18^ To achieve a shorter residence time for the gases in the filter, either 3, 2, or 1 filter candle was used, and the empty space for the removed filter candle was replaced with a closed metallic tube of the same size. In two experiments, a metal plate was added inside the filter vessel to still reduce the filter vessel volume from 17.6 to 13.5 L (Figure 3).

After the cyclones, hot vapors and gases were first cooled indirectly in a cold water-cooled heat exchanger (40 °C) after which vapors and gases were passed to an electrostatic precipitator (20 °C), where aerosols from the gases were recovered. From the electrostatic precipitator (ESP), the noncondensed water and light organics were led to two glycol coolers (−10 °C): first, a tube heat exchanger and second, a smaller tube heat exchanger filled with additional glass packings. The composition of the noncondensable gases was analyzed by gas chromatography. After the pyrolysis experiments, the collected char, bio-oil, and sand from the hot vapor filter were weighed. Most of the organics (≈95 wt %) were recovered in the water-cooled heat exchanger and electrostatic precipitator. The product recovered in the glycol cooler contained 80–85 wt % water. The liquid products recovered in the water-cooled heat exchanger and electrostatic precipitator were mixed before physicochemical characterization. The liquid recovered from the glycol coolers was treated separately. The liquid recovery system was rinsed after each experiment with a small amount of methanol to remove the condensed bio-oils from the walls of the condensers. The amount of bio-oil condensed on the walls was determined by evaporating the methanol from the washing liquid with a rotavapor, weighing the residue, and analyzing the water content of the residue. Product yields are reported on a dry basis from the starting feedstock. Pyrolytic water refers to water formed in the pyrolysis reactions (pyrolytic water = total water in liquid products – moisture of the feedstock).

## Pyrolysis Product Characterization

Physicochemical characterization of the FPBO was carried out by employing modified and validated standard methods.^24,25^ Water content was analyzed by Karl Fischer titration using a Metrohm 795 KFT Titrino titrator (ASTM E 203). Elemental composition analysis (CHN) was carried out using an Elementar VARIOMAX CHN analyzer (ASTM D 5291), and a higher heating value (HHV) was determined using an IKA Werke C 5000 Control calorimeter (DIN 51900). Carboxylic acid number (CAN) was determined with a 785 DMP Titrino analyzer (ASTM D 664), and the micro carbon residue (MCR) was analyzed using an Alcor Micro Carbon Residue Tester (ASTM D 4530). The ash content of the liquid was further determined by combusting the residue from the MCR determination in a muffle furnace at 775 °C. Carbonyls were analyzed by titration. The method used the reaction between hydroxylamine hydrochloride and pyridine to determine more than 30 aliphatic, alicyclic, and aromatic aldehydes and ketones.^26^ The chemical composition of the bio-oil was determined with the solvent fractionation scheme. In this method, bio-oil is first divided into a water-soluble (WS) and a water-insoluble (WIS) fraction by water extraction. The water-soluble fraction is further extracted with diethyl ether to an ether-soluble (ES) and an ether-insoluble (EIS, sugar-like material) fraction. The water-insoluble fraction is extracted with dichloromethane (DCM) to a DCM-soluble fraction containing low molecular mass (LMM) lignin and a DCM-insoluble fraction containing high molecular mass (HMM) lignin. The LMM fraction contains poorly water-soluble lignin monomers and dimers (MM = 400 Da) and extractives, while the HMM fraction contains powder-like high molecular mass (MM = 1050 Da) lignin-derived material and solids.^27,28^

## Results and Discussion

### Commissioning Tests of the Hot Vapor Filter with Stem Chips

Hot vapor filter was commissioned by testing different face velocities, i.e., number of filters (1–3), particle sizes of sand (0.2–1.0 mm), sand flows (1.5–3.0 kg/h), and filter temperatures (365–445 °C). Stem chips with an ash content of 0.2 wt % was used as feedstock. Gas residence time was kept constant in all tests, and two pyrolysis temperatures, 480 and 500 °C, were employed. Two cyclones were used before the hot gas filter in all tests to remove the main part of the char.

**Figure 4:**
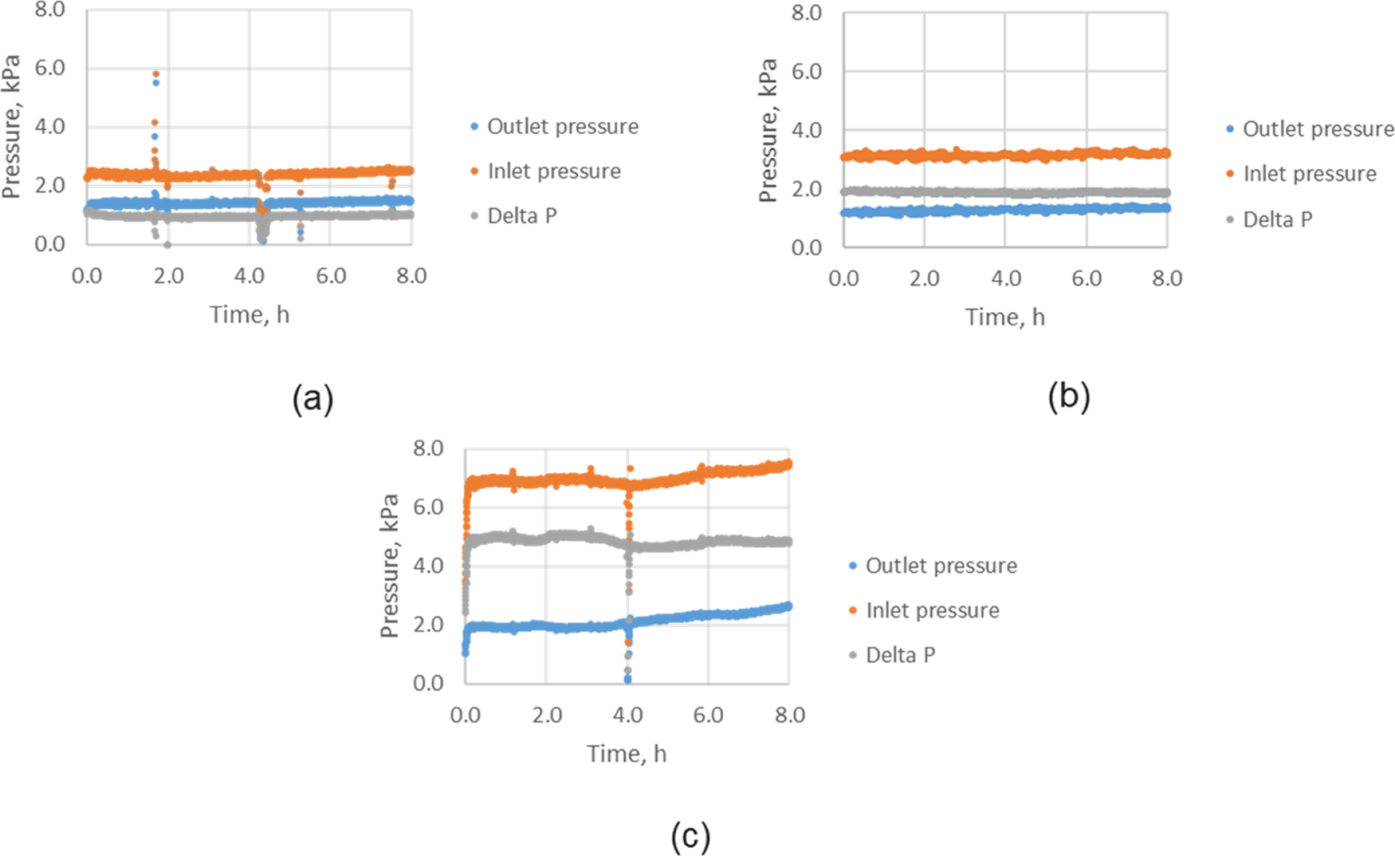
Inlet, outlet, and differential pressures of the hot filter during (a) 3 filters, run 3, (b) 2 filters run 4, and (c) 1 filter run 5.

The commissioning tests were carried out successfully without any major increase in pressure drop across the filters during the 8 h of operation (Figure 4). From the filter pressure profiles presented in Figure 4, it can also be seen that the reduction of filter elements from 3 to 2, i.e., increasing the face velocity by 50 vol %, roughly doubled the pressure drop across the filters. After the tests, the filter vessel was opened for inspection. The filter elements had changed their color from white to black, but no filter cake could be observed on the filter candles. The duration of the test was too short to assess any significant attrition or erosion of the filter elements. The new filter design with sand granules flowing around the filter candles seems to continuously remove the filter cake and prevent a pressure increase across the filters.

Compared to the experiments carried out without the filter, the reduction in organic liquid yield by approximately 5–10 wt % was seen, when the filter was connected to the unit. This is significantly lower compared to the 10–30 wt % loss reported for barrier filters in the literature.^16^ The reduction in organic liquid yield was more severe at higher filtration temperatures. As the filtration temperature was increased from 365 to 445 °C, the organic liquid yield was decreased from 56 to 42 wt % (Table 3). Cyclone char yield and reaction water remained roughly the same during these runs, ranging from 13 to 15 wt % for char and 10–12 wt % for water. Elemental analysis was done on filter sand to measure char deposition during filtration runs. As shown in Table 3, carbon yield in sand was close to 1% in all tests. On the other hand, pyrolytic gas yields displayed a consistent increase from 12 to 20 wt %, as the filtration temperature increased from 365 to 445 °C. The reduction in bio-oil yields and the increase in gas yields were attributed to secondary cracking of vapors at high filtration temperatures. To reduce the loss of oil yield, the lowest possible filtration temperature should be used, where condensation of the vapors would not occur. According to Scahill et al.,^18^ lower temperatures than 370–390 °C should be avoided except for the cases of relatively high carrier gas flow rates.^18^ The 365 °C used in experiment number 12 was already very close to this temperature range, and therefore, any further experiments were not carried out to optimize the filter temperature. The influence of face velocity, sand particle size, and sand flow was also tested in a few experiments, but these did not have any clear impact on the product distribution.

**Table 3:**
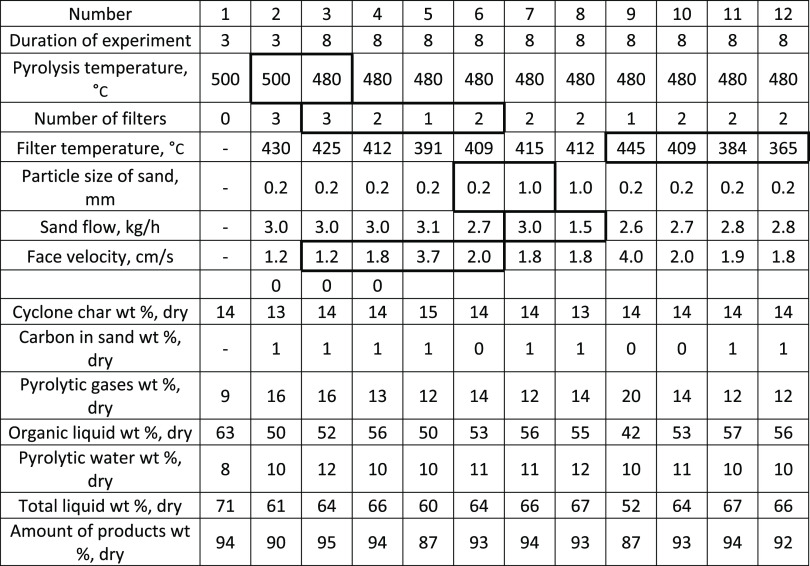
Influence of Pyrolysis Temperature, Face Velocity, Sand Particle Size, Sand Flow, and Filter Temperature on Product Yields with Stem Chips

**Figure 5:**
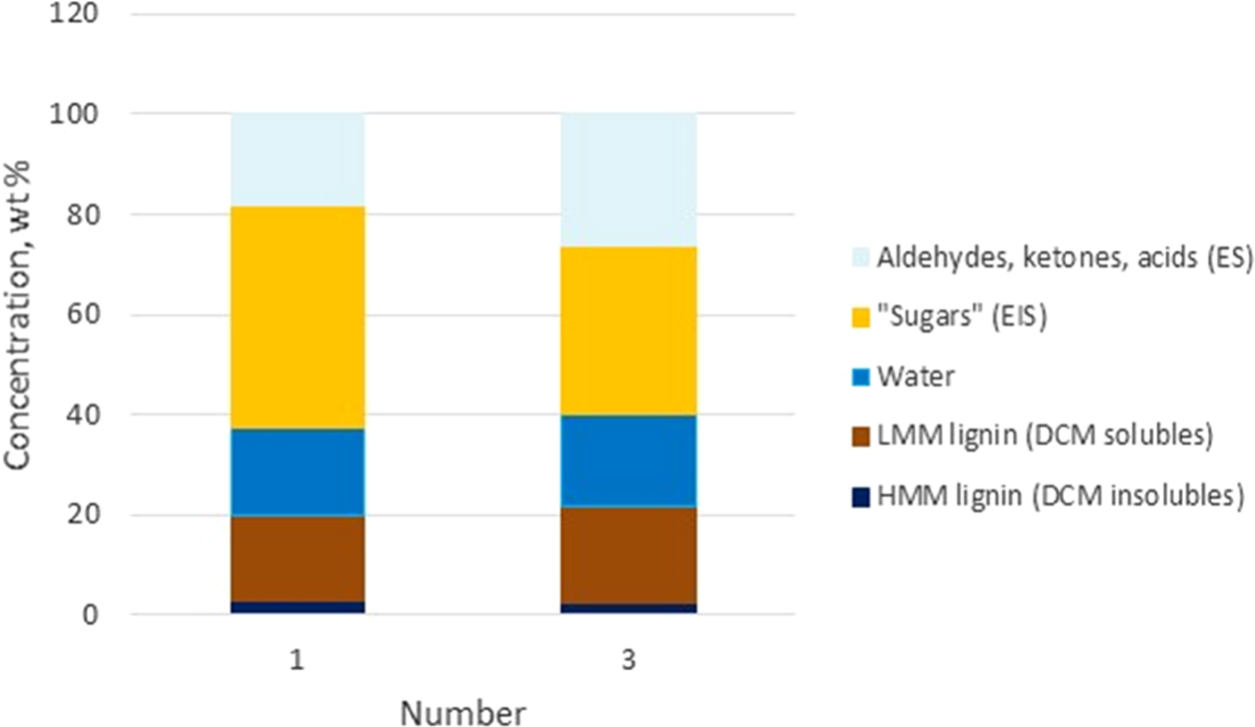
Chemical composition of the bio-oil produced without a filter (number 1) and with a hot vapor filter (number 3).

Filtered bio-oil exhibited higher water content than the unfiltered one. As shown in Table 4, water contents during runs were mostly between 15.0 and 20.2 wt %, while the unfiltered bio-oil from run 1 had a water content of 17.4 wt %. The higher water content after filtration is caused by the decrease in organic liquid yield. To get a better understanding of the effect of the filter on bio-oil composition, the reference oil produced without the filter (experiment number 1) and a filtered bio-oil (experiment number 3) were characterized for chemical composition by the solvent fractionation scheme (Figure 5).^27,28^ Based on the analyses, no significant difference was seen in the lignin fraction between the unfiltered and filtered oil, while the sugar fraction significantly decreased after filtration. Similar reduction in sugar concentrations has been reported between de-ashed and untreated feedstocks.^29^ It is possible that the longer residence time in the filter vessel in combination with the catalytic effect of the metals in the feedstock promoted the decomposition reactions of the polysaccharides. The lower sugar concentration in bio-oils from hot vapor filtration reduced also to some extent the oxygen content of the bio-oil. Other fuel properties including MCR, CAN, and carbonyl content were not affected by the filtration. Ash content was already very low for the unfiltered bio-oil, and therefore, detailed metal analyses were needed to see the real impact of the filter on metal removal.

**Table 4:**
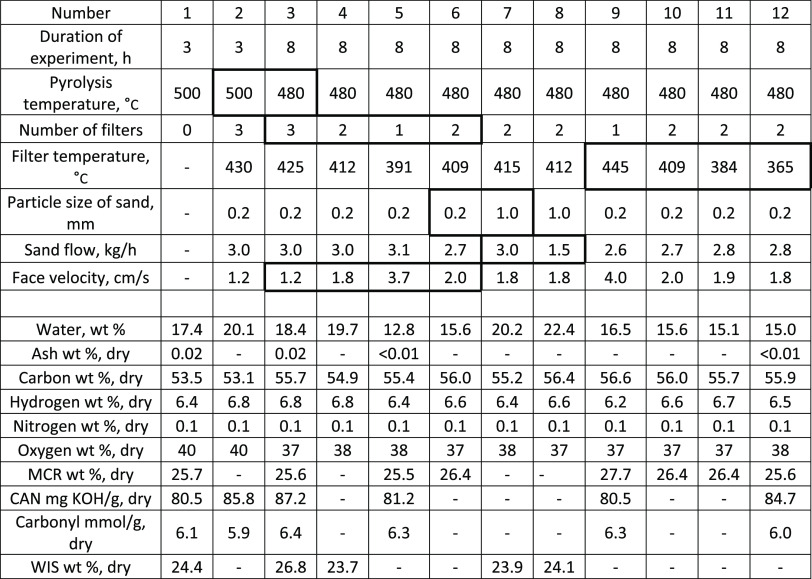
Influence of Pyrolysis Temperature, Face Velocity, Sand Particle Size, Sand Flow, and Filter Temperature on Liquid Properties with Stem Chips

One filtration experiment was carried out without the cyclones to investigate if the hot vapor filter can be located directly after the reactor. As expected, more organic liquid was lost when all char entered the filter vessel (Table 5). The reduction in the organic liquid yield was caused by more cracking due to higher concentrations of ash in the filter vessel, which increased the gas yield from 12 to 18 wt %. The organic liquid yield was reduced from 57 to 45 wt % and solely the gas formation does not fully explain the cracking mechanism. Probably, some coke formation also took place inside the filter vessel, but because of the large amount of sand used in the experiment, this coke could not be accurately measured. Separation of char with cyclones is therefore highly recommended before filtration to reduce the loss of product.

**Table 5:** Product Yields from Pyrolysis Test with and without Char Removal before the Hot Vapor Filter

number	1	11	13
char removal with cyclones	yes	yes	no
duration of the experiment	3	8	8
pyrolysis temperature (°C)	500	480	480
number of filters	0	2	2
filter temperature (°C)		384	380
particle size of sand (mm)		0.2	0.2
sand flow (kg/h)		2.8	2.8
face velocity (cm/s)		1.9	1.8
cyclone char wt %, dry	14	14	1
carbon in sand wt %, dry		1	2
pyrolytic gases wt %, dry	9	12	18
organic liquid wt %, dry	63	57	45
pyrolytic water wt %, dry	8	10	13
total liquid wt %, dry	71	67	57
amount of products wt %, dry	94	94	78

Alkali and alkaline earth metals alongside with some heavy metals were analyzed from some of the bio-oils to see the real impact of the filter on metal removal (Table 6). The unfiltered bio-oil from stem chips was already very clean with potassium, magnesium, sodium, and calcium concentrations below 10 mg/kg, which is the detection limit for the metals. The metal removal could therefore not be further improved in experiment number 4 using the hot vapor filter. Experiment number 13 was carried out without the cyclones, and one would have expected that if the filter would not have worked properly, the metal content in this oil would have been much higher. Considering both the analytical limitations and the possibility for bio-oil contamination by leaching from the liquid recovery system, it is difficult to make any conclusion based on the experiments with stem chips on how much of the metals can be removed. Further tests with more contaminated feedstocks were needed to evaluate the filter performance.

**Table 6:** Metal Analysis of Unfiltered and Filtered Bio-Oil with Stem Chips

number	1	4	13
char removal with cyclones	yes	yes	no
duration of the experiment (h)	3	8	8
pyrolysis temperature (°C)	500	480	480
number of filters	0	2	2
filter temperature (°C)		412	380
particle size of sand (mm)		0.2	0.2
sand flow (kg/h)		3.0	2.8
face velocity (cm/s)		1.8	1.8
Na mg/kg, dry	<10	<10	<10
K mg/kg, dry	<10	27	<10
Mg mg/kg, dry	<10	<10	<5
Ca mg/kg, dry	<10	<10	8
Cr mg/kg, dry	<0.5	<0.5	<1
Mn mg/kg, dry	<0.5	<0.5	<1
Fe mg/kg, dry	1.6	1.6	<5
Cu mg/kg, dry	<0.5	<0.5	<1
Zn mg/kg, dry	<0.5	<0.5	2
Si mg/kg, dry	17	23	181
Pb mg/kg, dry	<0.5	<0.5	<10
P mg/kg, dry	<10	<10	<5
S mg/kg, dry	22	22	<50
Cl mg/kg, dry	50	80	158

### Hot Vapor Filtration Tests with Contaminated Wood and Forest Residue

Hot vapor filtration experiments with contaminated wood and forest residue were carried out to investigate the performance of the filter with more sustainable feedstocks containing higher amounts of impurities. The experiments with contaminated wood were carried out by varying the filter temperatures (450, 400, 360 °C) and residence time (6–9 s) of the gases and vapors in the filter vessel. The residence time was varied using either 3, 2, or 1 filter candle and replacing the empty space for the removed filter candle with a closed metallic tube of the same size. In experiments 19 and 21, an extra plate was added to the filter vessel to still reduce the residence time.

All tests were carried out successfully without any pressure increase across the filter during the 6 h filter experiments. The results (Table 7) were in line with those obtained with stem chips. The highest organic liquid yield (48 wt %) from contaminated wood was obtained with the lowest filtration temperature (359 °C). The next step was to use this temperature and vary the residence time of vapors and gases. In our system, only small reductions of the residence times (9–6 s) were possible without building a totally new filter vessel. Already these small changes increased the organic liquid yield from 48 to 52 wt %. These results show that there is still a possibility for yield improvements by reducing the volume of the filter vessel. The experiments worked also well with only one filter candle, and therefore, higher face velocities might also be possible. Contaminated wood used in the experiments had only slightly higher ash content (0.8 wt %) compared to stem chips, and therefore, the performance of the filter for metal removal is still vague. To get a better understanding of the filter performance, the last condition was repeated for the forest residue. Forest residue had an ash content of 5.4 wt %, which reduced the organic liquid yield even more after filtration (46 wt %) compared to the reference test without a filter (52 wt %). With forest residue, more metals will escape the cyclones and enter the filter vessel, which, during the long residence time, catalyze the cracking of the organic molecules. The reduction of the residence time and the fast removal of the sand from the filter vessel with the metals might then be more important with residual feedstocks compared to stem wood.

**Table 7:**
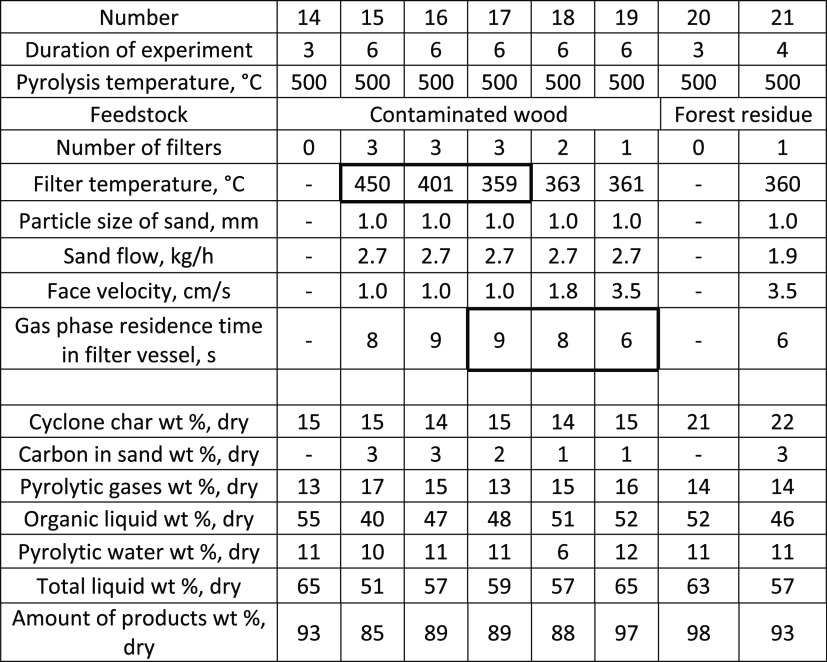
Influence of Pyrolysis Temperature and Residence Time on Product Yield with Contaminated Wood and Forest Residue

The physical and chemical properties analyzed for the contaminated wood and forest residue bio-oils are presented in Table 8. Similar to the pyrolysis experiments carried out with stem chips, higher water content was measured for the filtered bio-oil compared to the reference bio-oil without filtration. The filtration cracked more of the sugar-derived compounds, which can be seen as a decrease in oxygen content, MCR, and carbonyl content as well as an increase in CAN.

**Table 8:**
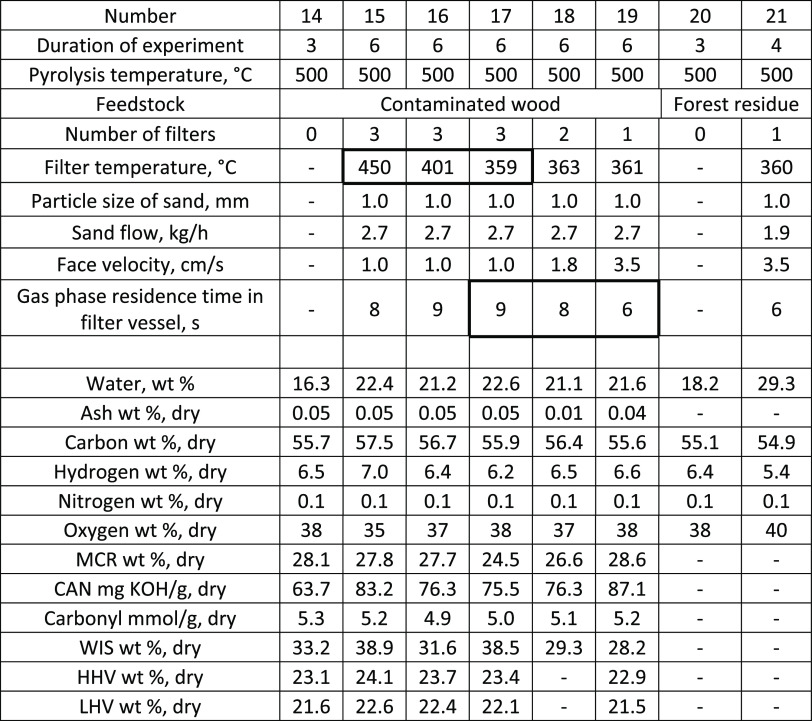
Physicochemical Properties for the Bio-Oils Produced from Contaminated Wood and Forest Residue

Alkali and alkaline earth metals alongside some heavy metals were analyzed from some of the bio-oils produced from contaminated wood and forest residue (Table 9). Like the metal analyses for the unfiltered oil from stem chips, also the unfiltered bio-oils from contaminated wood and forest residue possess very low metal contents. Due to the analytical limitations in detection of low concentrations of metals from the bio-oils, only some minor reduction in metal content was seen between the filtered and unfiltered bio-oil. The overall metal removal was improved from 97 to 98 wt % for contaminated wood using the hot vapor filter, while no change was seen with forest residue. The bench-scale system used in the tests had small cyclones, which already removed very efficiently the solids from the vapors before the hot gas filter. The real benefit of the filter in solid removal and continuous operation without pressure increase can therefore be seen first on a pilot scale, where solid removal is not as efficient. A possible location for the filter in a large-scale pyrolysis plant is after the first cyclone and before the liquid recovery system. Regenerated sand from the sand heater should be used after mild cooling to continuously remove the filter cake from the filter candles. The sand flow should also be minimized to reduce the overall cost of filtration.

**Table 9:**
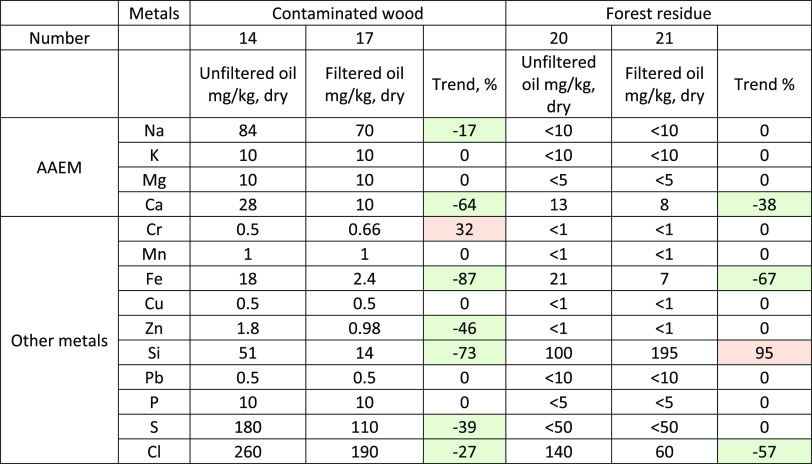
Metal Analysis of Unfiltered and Filtered Bio-Oils from Contaminated Wood and Forest Residue

## Conclusions

A new filter design, combining the barrier and moving bed filter, was successfully tested on a bench scale with stem chips, contaminated wood, and forest residue. No significant increase in pressure drop across the filter was seen during the 6–8 h of operation, proving that the new filter design with sand granules flowing around the filter candles continuously removes the filter cake.

The filter was tested at different face velocities by changing the number of filters from 1 to 3, particle size of sand (0.2–1 mm), sand flow rates (1.5–3 kg/h), filter temperatures (360–450 °C), and residence times of gases and vapors in the filter vessel (6–9 s). Temperature and residence time affected mostly the organic liquid yield. Using the lowest filtration temperature (360 °C) and shortest residence time (6 s) for contaminated wood, the organic liquid yield was reduced only from 55 wt % for the reference oil without a filter to 52 wt % for the filtered oil. Small variations in residence times were only possible with the current filter vessel, and therefore, there are possibilities for yield improvements by designing a smaller filter vessel with only one filter candle. One experiment was also carried out without the cyclones to investigate if the hot vapor filter can be located directly after the reactor. The entrainment of the char and metals into the filter vessel affected negatively the organic liquid yield, and therefore, efficient removal of solids with cyclones is still needed before the filtration.

The products from the filtration experiments were also analyzed for physical and chemical properties. The polysaccharides were cracked more during the filtration, resulting in a bio-oil with slightly reduced oxygen content, MCR, and carbonyl content but with an increased acidity (CAN). The bio-oils produced without filtration from stem chips, contaminated wood, and forest residue were already very clean with metal concentrations close to the detection limits of the analytical procedure. It was therefore impossible to see any further improvements in metal removal after filtration. The bench-scale system used in the experiments had small cyclones, which already removed very efficiently the solids from the vapors. The actual benefit of the filter can therefore first be seen on a pilot scale with less efficient cyclones.
